# Predicting treatment plan approval probability for high-dose-rate brachytherapy of cervical cancer using adversarial deep learning

**DOI:** 10.1088/1361-6560/ad3880

**Published:** 2024-04-17

**Authors:** Yin Gao, Yesenia Gonzalez, Chika Nwachukwu, Kevin Albuquerque, Xun Jia

**Affiliations:** 1 Department of Radiation Oncology, University of Texas Southwestern Medical Center, Dallas, TX, United States of America; 2 Department of Radiation Oncology and Molecular Radiation Sciences, Johns Hopkins University, Baltimore, MD, United States of America

**Keywords:** deep learning, high dose rate brachytherapy, treatment planning, plan approval probability

## Abstract

*Objective.* Predicting the probability of having the plan approved by the physician is important for automatic treatment planning. Driven by the mathematical foundation of deep learning that can use a deep neural network to represent functions accurately and flexibly, we developed a deep-learning framework that learns the probability of plan approval for cervical cancer high-dose-rate brachytherapy (HDRBT). *Approach.* The system consisted of a dose prediction network (DPN) and a plan-approval probability network (PPN). DPN predicts organs at risk (OAR) *D*_2*cc*_ and CTV *D*_90%_ of the current fraction from the patient’s current anatomy and prescription dose of HDRBT. PPN outputs the probability of a given plan being acceptable to the physician based on the patients anatomy and the total dose combining HDRBT and external beam radiotherapy sessions. Training of the networks was achieved by first training them separately for a good initialization, and then jointly via an adversarial process. We collected approved treatment plans of 248 treatment fractions from 63 patients. Among them, 216 plans from 54 patients were employed in a four-fold cross validation study, and the remaining 32 plans from other 9 patients were saved for independent testing. *Main results.* DPN predicted equivalent dose of 2 Gy for bladder, rectum, sigmoid *D*_2*cc*_ and CTV *D*_90%_ with a relative error of 11.51% ± 6.92%, 8.23% ± 5.75%, 7.12% ± 6.00%, and 10.16% ± 10.42%, respectively. In a task that differentiates clinically approved plans and disapproved plans generated by perturbing doses in ground truth approved plans by 20%, PPN achieved accuracy, sensitivity, specificity, and area under the curve 0.70, 0.74, 0.65, and 0.74. *Significance.* We demonstrated the feasibility of developing a novel deep-learning framework that predicts a probability of plan approval for HDRBT of cervical cancer, which is an essential component in automatic treatment planning.

## 1. Introduction

Treatment planning is one of the most important steps for the success of cancer radiation therapy. The treatment planning problems in modern radiation therapy techniques, e.g., intensity modulated radiation therapy, volumetric modulated arc therapy, and high-dose-rate brachytherapy (HDRBT) are often solved via an optimization approach. Generally speaking, the objective function of the optimization problem is defined by a number of parameters, such as the specific variables defining different dosimetric constraints and their relative weights. Once a planner defines these parameters, and hence the objective function, the optimization engine in a treatment planning system (TPS) is launched to solve the optimization problem and generate a solution. The planner repeatedly adjusts parameters to refine the objective function and improve the resulting plan quality. In this process, the planer often consults with the physician about plan quality, who determines the acceptance of the resulting plan. This process is apparently tedious and time-consuming. The quality of the resulting plan depends on several factors such as the experience of the planner, the available time for planning, and interactions between the planner and the physician. This is particularly a problem for time-critical scenarios, such as in HDRBT (Mayadev *et al* 2014) or adaptive radiotherapy (Yan *et al* 1997, Li *et al* 2013), where the time available for treatment planning is limited. Hence, automation is highly desired to improve this treatment planning process.

Over the years, numerous methods have been developed to guide the optimization engine of treatment planning to automatically generate high-quality plans, ranging from classical optimization-based approaches (Xing *et al* 1999, Wu and Zhu 2001, Lu *et al* 2007) to recent reinforcement learning approaches (Shen *et al* 2019, 2020a, 2021a, 2021b, Zhang *et al* 2021, Pu *et al* 2022). Yet, a critical component, to quantify the quality of a plan in a mathematical form remains the central question for automatic treatment planning. The plan quality can be judged by meeting certain requirements, such as dose-volume constraints of the tumor target and organs at risk (Miften *et al* 2004, Leung *et al* 2007). More importantly, since it is the physicians preference that decides the appropriate trade-offs between different criteria, it is of critical importance to predict the physicians preference in plan approval to enable an effective automatic treatment planning process. In the past, there have been extensive studies in this direction. For example, Delgado *et al* (2018) developed an analytical hierarchy process that was able to select a treatment plan from a set of options according to the preferences of the respondent. Knowledge-based planning was successfully developed to extract ‘knowledge’ about physician-preferred dose distributions by analyzing a library of previously approved plans (Tol *et al* 2015, Ge and Wu 2019). With the recent developments in deep learning (Sahiner *et al* 2019, Shen *et al* 2020b), there has been a plethora of research continuing in this direction. Studies have reported the feasibility of predicting the optimal dose distribution based on patient-specific anatomy using deep learning (Chen *et al* 2019, Nguyen *et al* 2019).

For automatic treatment planning, it is desired to have a numerical metric to directly predict the probability of plan approval. Such a metric could take the form of a scalar value as a function of the input plan and patient anatomy, representing the probability of having the plan acceptable to the physician. The metric can be built by analyzing the treatment plans available from previous patients. Once established, it can be used to guide an automatic treatment planning tool to achieve a plan with the highest metric value. Yet, there are two practical challenges, when constructing a metric like this. First, the specific function form representing the chance to approve a plan can be very complex, and from a practical perspective, we must assume a specific function form to allow the determination of the function through a fitting process using existing patient cases. Properly choosing the function form is important to ensure that the result is not limited by this choice. Previously, Willoughby *et al* (1996) employed an artificial neural network to establish a correlation between the dosimetric measures of the plans with the quality scores of the physician’s plan. With the advance of deep learning, deep neural networks (DNN) may be used to build this plan approval prediction function because of their large capacities and flexibility to approximate function forms Our recent work demonstrated the feasibility of using a DNN to represent physicians preference in external beam radiation therapy (EBRT) setting using planning structure images and a dose distribution (Gao *et al* 2022). Yet the idea in the HDRBT domain has not been explored. The second challenge comes from the fact that only previously approved plans are usually available, whereas unapproved plans generated during the planning process have been discarded. This fact prevents us from building a model to predict plan approval via a straightforward classification point of view by generating a classification function to discriminate between approved and unapproved plans.

In this paper, we will present our recent study that models the probability of treatment plan approval for HDRBT of cervical cancer treated with a tandem and ovoid applicator (T&O). We will use a DNN as a generic, yet flexible, function approximator to describe the probability of plan approval as a function of key dosimetric parameters of a plan and patient anatomy. We will employ an adversarial training scheme (Kurakin *et al* 2016, Mahmood *et al* 2019) to develop the prediction model of plan approval using existing approved plans. Different from the plan approval problem in EBRT, one unique feature in HDRBT of cervical cancer is that a physician considers the total dose of the entire treatment course for plan approval, including that of EBRT, delivered fractions, the current fraction seeking approval, and expected remaining fractions. Hence, the unique contribution of this study also included the development of a network structure to incorporate this feature.

## 2. Methods

### 2.1. Overall idea and feature representations of dose and anatomy

The general goal of this work is to predict the probability to approve HDRBT plan for a patient. Specifically, we will build a DNN model, called plan-approval probability network (PPN). PPN has two inputs. The first is a feature representation of the plan seeking approval, while the second is that of the patient’s anatomy relative to the applicator. We extract feature representations of the plan dose and patient anatomy, instead of directly using them as input, to highlight key information about the plan and anatomy based on our prior knowledge. This approach is expected to reduce the complexity of model training and the required size of the data. The output of the PPN model is a number in [0, 1] representing the probability that the plan is approved by the physician.

For the representation of the plan seeking approval, we considered four dosimetric measures to represent its quality, including *D*_90%_ of the high-risk clinical planning target (CTV), *D*_2*cc*_ of three organs at risk (OARs): bladder, rectum, and sigmoid colon. A physician focuses primarily on these four dosimetric measures to examine plan quality, because they are relevant to the outcome of the treatment in HDRBT of cervical cancer (Georg *et al* 2012, Mazeron *et al* 2016, Tanderup *et al* 2016). These quantities were expressed in the form of an equivalent dose of 2 Gy (EQD2) (Hall *et al* 2006) in the rest of this article unless otherwise stated. When calculating the EQD2 from physical doses, the *α*/*β* ratios of 3 Gy for OARs and 10 Gy for CTV were used.

Unlike the plan approval problem in EBRT, one issue in HDRBT is that a physician would, in fact, consider the total EQD2 of the entire treatment course. The total EQD2 includes that of EBRT, HDRBT fractions delivered already, and the current plan. It was calculated by assuming any untreated future HDRBT fractions would give the average dose in those fractions already delivered. Suppose that we are considering the *i*th fraction of HDRBT treatment in a course with total *N* fractions. Let us denote the four dosimetric measures of the current plan as *D* ^*i*^, those of previously delivered plans as *D* ^*i*−^, and those from EBRT as *D* ^EBRT^. The total dose *D* ^*Ti*^ of the entire course is calculated as

\begin{eqnarray*}{D}^{{Ti}}\equiv F({D}^{i})=\displaystyle \frac{N}{i}{D}^{i}+\displaystyle \frac{N}{i}{D}^{i-}+{D}^{\mathrm{EBRT}},\end{eqnarray*}

where the factor *N*/*i* indicates that we extrapolate the mean HDRBT dose delivered up to (including) the current fraction to all the HDRBT fractions. *D* ^*i*^ was converted to *D* ^*Ti*^ using the function *F*(.) in equation (1) before being input to the network.

**Figure 1.**
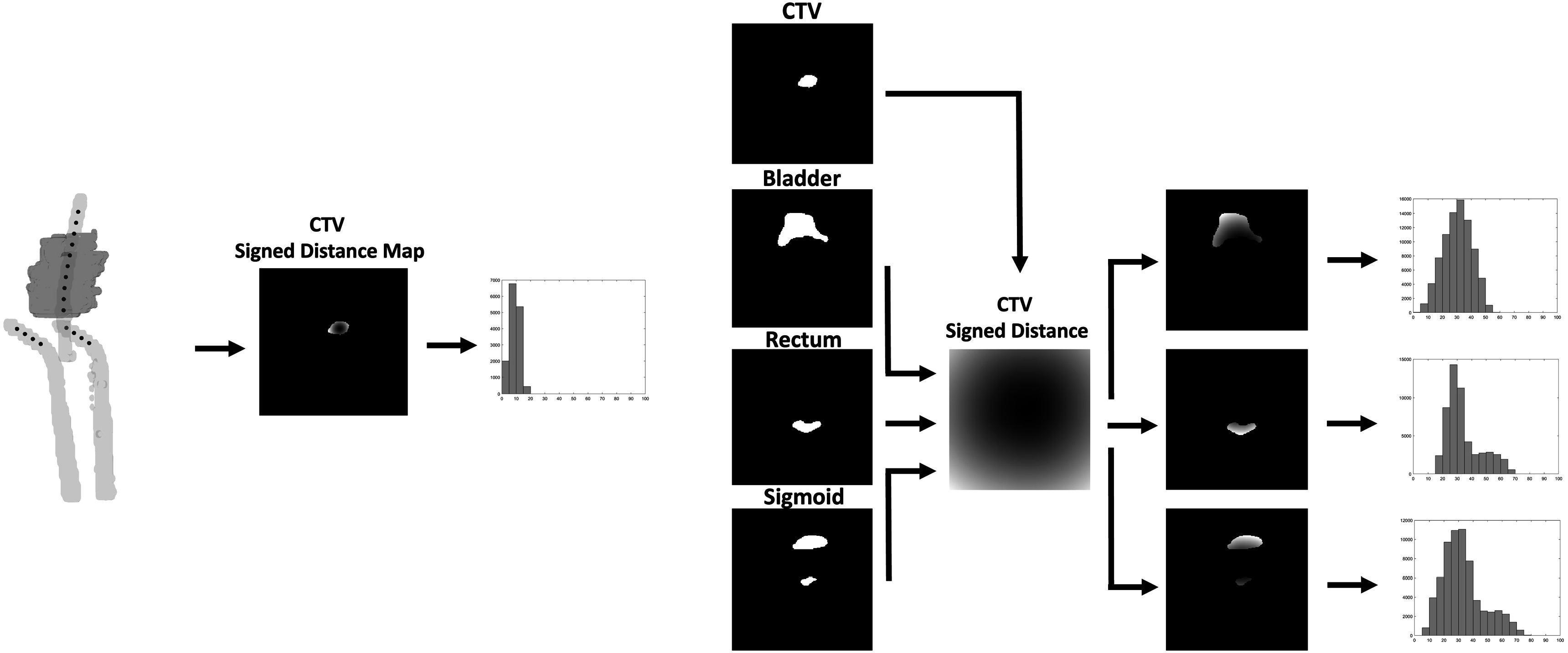
Outline of creation of distance histograms representing patient anatomy. (Left) CTV distance map was created representing the closest distance of each voxel in the CTV (shown in blue) to any given source located within the applicators (black *). (Right) For each OAR, a distance map was created representing the closest distance of each voxel in the OAR to the CTV boundary. All distance maps were then binned to create the distance histograms.

We further denote the anatomy of the patient relative to the applicator in the *i*th fraction as *A* ^*i*^, which was the second input to the PPN. In principle, a DNN model can be trained to take anatomy images, e.g. binary contour images, directly as input (Chen *et al* 2019, Nguyen *et al* 2019). In this study, we modeled the anatomy *A* ^*i*^ as a set of four distance histograms for CTV and three OARs. Distance histogram has been found to be a useful tool in describing anatomy geometry in plan quality analysis (Ge and Wu 2019). As such, for the CTV, we calculated the shortest distance of each voxel within it to the dwell positions in the plan and then generated its distance histogram by binning the list of distance values into 20 equal-sized bins ranging from 0 to 100 mm. For OARs, we first created a distance map to the CTV boundary, a volumetric image representing the shortest distance from each voxel to the CTV boundary. For each OAR, we singled out those distance map values for voxels inside the OAR and then created a distance histogram in the same way as for the CTV distance histogram. Figure 1 illustrates this procedure. The four vectors, each of the length of *n* = 20 elements representing the distance histogram of a structure, were input to the PPN model.

### 2.2. Network structures

**Figure 2.**
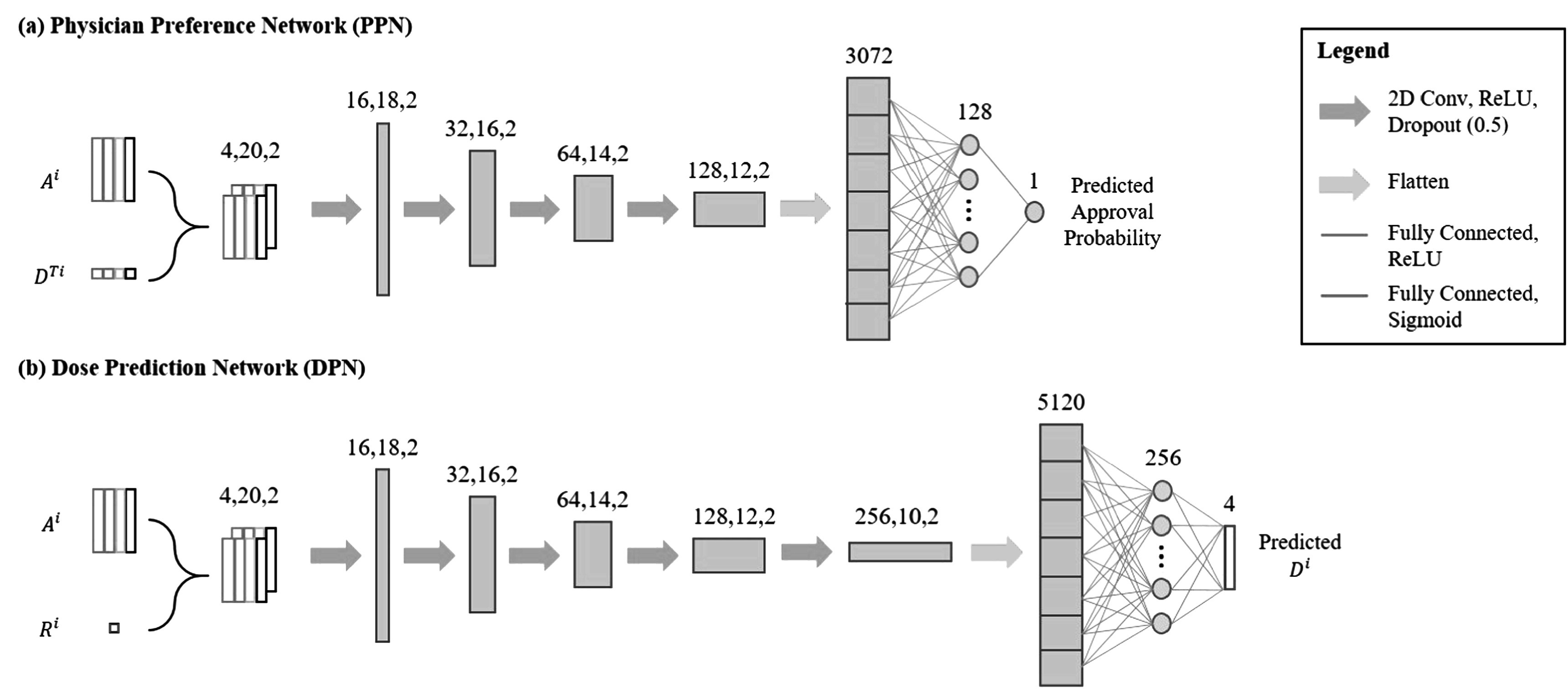
Network architecture of the (a) PPN and (b) DPN. The input dimension to each layer is shown above the layer.

With the dosimetric measures of a plan and the representation of the patient’s anatomy relative to the applicator, we formed the PPN shown in figure 2(a). The distance histograms *A* ^*i*^ were in the form of a matrix 4 × *n*, where the four sections correspond to the four structures, each with a histogram of a length *n* = 20. PPN also considered the total EQD2 of four structures *D* ^*Ti*^ in the network input. The vector with four elements was replicated *n* times to a matrix with a size of 4 × *n* to be combined with *A* ^*i*^. The inputs were first processed by four 2D convolutional layers, each followed by a Leaky Rectified Linear Unit (LeakyReLU) and a dropout layer, prior to a flattening layer. Finally, the data was processed by two dense layers followed by a sigmoid activation function, yielding a single value in [0, 1] representing the probability of having the plan approved by the physician given the plan and anatomical information.

Training PPN would be straightforward if there were both approved and unapproved plans for each patient case. In this situation, the PPN could be simply trained with paired input-output data to solve a classification problem. However, the practical challenge is that only previously approved plans are stored in clinical practice, whereas unapproved plans during the treatment planning process have already been discarded. To overcome this challenge, we employed an adversarial training scheme (Kurakin *et al* 2016, Mahmood *et al* 2019). Specifically, a second network, dose prediction network (DPN), was constructed, which served the purpose of predicting the four EQD2 values *D* ^*i*^ in the *i*th fraction of HDRBT treatment based on the patients anatomy *A* ^*i*^ and the prescription dose of HDBRT *R* ^*i*^. We designed the DPN structure as shown in figure 2(b). The single number of prescription dose was replicated 4 × *n* times to form a matrix of this size and combined with *A* ^*i*^ as the input to the network. The network followed the same structure and convolution operations as the PPN except for the last layer. After a LeakyReLU activation function in the final fully connected layer, DPN predicted EQD2 of four structures for the current fraction based on four inputs. Note that the output of DPN was converted to total HDRBT dose via equation (1) before being input to PPN.

### 2.3. Adversarial training strategy

In the adversarial training scheme, DPN and PPN were jointly developed. DPN was trained to predict the dosimetric measures as accurately as possible, while PPN was trained to differentiate the ground truth dosimetric measures from those generated by the DPN, which may not be accurate during the training process. Let us denote the data as a quadruplet (*A*_*j*_, *D*_*j*_, *R*_*j*_, *Y*_*j*_) for case *j*, where *A*, *D*, and *R* stand for the patient anatomy representation, the plan dose representation, and prescription dose of HDRBT, and *Y* = 0 or 1 being the plan approval status (1 means approval and 0 otherwise). Here the index *j* covers different patient cases and fractions within a treatment course. Note that for data available to develop our model, *Y* = 1 as all data were previously approved plans. Denote the two networks as DPN(*R*, *A*∣*θ*_DPN_) and PPN(*D*, *A*∣*θ*_PPN_), respectively. *θ*_DPN_ and *θ*_PPN_ are the network parameters of the two networks to be determined via the network training process. The loss function and the overall optimization problem of this study can be formulated as a min-max problem:

\begin{eqnarray*}\begin{array}{l}\mathop{\min }\limits_{{\theta }_{\mathrm{DPN}}}\mathop{\max }\limits_{{\theta }_{\mathrm{PPN}}}L({\theta }_{{DPN}},{\theta }_{{PPN}})=\mathop{\min }\limits_{{\theta }_{\mathrm{DPN}}}\mathop{\max }\limits_{{\theta }_{\mathrm{PPN}}}{{\mathrm{E}}}_{j\in {Tr}}\left[| \mathrm{PPN}(F({D}_{j}),{A}_{j}| {\theta }_{\mathrm{PPN}}){| }^{2}\right]\\ \quad +{E}_{j\in {Tr}}\left[| 1-\mathrm{PPN}(F(\mathrm{DPN}({R}_{j},{A}_{j}| {\theta }_{\mathrm{DPN}})),{A}_{j}| {\theta }_{\mathrm{PPN}}){| }^{2}\right],\end{array}\end{eqnarray*}

where ${{\mathrm{E}}}_{j\in {Tr}}\left[.\right]$ denotes expectation over the training dataset *Tr*. The function *F*(.) defined in equation (1) converts fractional dose into total EQD2. Minimizing the loss function applied to training the DPN, which enforced the accuracy of generated plan DPN(*R*_*j*_, *A*_*j*_∣*θ*_DPN_), because the minimization of the second term in the loss function would favor a plan dose DPN(*R*_*j*_, *A*_*j*_∣*θ*_DPN_) for the anatomy *A*_*j*_ being approved. Meanwhile, maximizing the loss function would favor a PPN that can differentiate real plans from plans generated by the DPN, namely PPN(*F*(*D*_*j*_), *A*_*j*_∣*θ*_PPN_) ∼ 1, while PPN(*F*(DPN(*R*_*j*_, *A*_*j*_∣*θ*_DPN_)), *A*_*j*_∣*θ*_PPN_) ∼ 0.

Training the two networks directly from scratch is challenging, as the initial condition is critical for the highly non-convex min-max optimization problem. Hence, we employed a strategy to first individually train the two networks to obtain a reasonable initial solution, before jointly training the two. In this step, the DPN was trained to predict EQD2 from the patient-specific anatomy in the HDRBT plan, as for each patient case the ground truth dose for the approved plan was known. As such, we solved the following optimization problem

\begin{eqnarray*}\mathop{\min }\limits_{{\theta }_{{DPN}}}{E}_{j\in {Tr}}\left[| \mathrm{DPN}({R}_{j},{A}_{j}| {\theta }_{\mathrm{DPN}})-{D}_{j}{| }^{2}\right].\end{eqnarray*}

To initially train the PPN, we purposely generated a number of plans considered as unapproved ones by perturbing each approved plans EQD2 randomly. Specifically, for the case (*A*_*j*_, *D*_*j*_, *R*_*j*_, *Y*_*j*_ = 1), we perturbed the EQD2 by a random magnitude ranging from 5% to 25% in the unfavorable direction. This generated a new plan with dose ${D}_{j}^{\prime} $ that was assumed to be associated with the label ${Y}_{j}^{\prime} =0$. Training PPN was then achieved by solving the optimization problem

\begin{eqnarray*}\mathop{\min }\limits_{{\theta }_{{PPN}}}{E}_{j\in {Tr}\cup {Tr}^{\prime} }\left[| \mathrm{PPN}(F({D}_{j}),{A}_{j}| {\theta }_{\mathrm{PPN}})-{Y}_{j}{| }^{2}\right],\end{eqnarray*}

where ${Tr}^{\prime} $ denotes the generated training dataset containing $({A}_{j},{D}_{j}^{\prime} ,{R}_{j},{Y}_{j}^{\prime} =0)$.

Once both networks were sufficiently pre-trained, they were jointly trained by alternatively solving the minimization and the maximization problems in equation (2) repeatedly. When solving one of the two sub-problems, the network that was not trained was ‘frozen’, while the other network was updated by the network training optimization algorithm.

### 2.4. Data preparation, implementation, and evaluations

All CT volumes were acquired from patients previously treated in the Department of Radiation Oncology at UT Southwestern Medical Center for locally advanced cervical cancer using HDRBT with T&O sets from Varian (Varian Medical System, Palo Alto, CA). The specific applicator setup, e.g. tandem angle and ovoid size were case dependent on specific patient anatomy. We obtained 248 unique plans including contours and dose distributions from 63 patients over at most 5 treatment fractions per patient. Each of the patients used in this study was treated by the same physician with an HDRBT prescription dose between 550 to 800 cGy per fraction. For each plan, the contours that were used in calculating distance histograms were manually delineated by a physician. The applicator was digitized manually by the planner and the plan was approved by the treating physician. These data were exported from the Eclipse BrachyVision TPS (Varian Medical System, Palo Alto, CA) in DICOM-RT format. We processed the contours in MATLAB to generate a binary volumetric image for each structure with a voxel size of 1 × 1 × 1 mm^3^, and subsequently to compute the distance histograms. Dose distributions were resampled to match this resolution.

Of this data set, we conducted a four-fold cross-validation study using 216 plans from 54 patients. Specifically, at each fold, 162 plans from 44 patients were used for model training, and 54 plans from 10 patients were for model validation. Among the four models, the best-performed model was advanced to the next stage for independent testing. 32 plans from the remaining 9 patients, which were not seen by the training and validation process, were used exclusively for testing.

All the computations related to network training and analysis were implemented using PyTorch on Python at the backend using two Nvidia RTX 5000 GPU cards. The optimization problems were solved via backpropagation using the Adam optimizer with L2 regularization. The initial learning rate was set to 5 × 10^−4^. We applied the ReduceLROnPlateau function in Pytorch, an adaptive learning rate algorithm to reduce the learning rate by 0.1 when validation loss has stopped improving for 5 epochs. Other parameters included the batch size of 4, the dropout rate of 0.5, the convolution kernel size of 3, and the LeakyReLU slop of 0.2. Hyperparameters in the network training process were manually fine-tuned to achieve the best performance for the corresponding validation data set. In the pre-training step, we trained PPN and DPN individually each for 100 epochs. Subsequently, the two networks were jointly trained for 100 epochs.

To demonstrate the performance of PPN, for each clinically approved plan, we created extra plans considered as unapproved by randomly perturbing the EDQ2 dose in HDRBT by ±5%, ±10%, ±15%, and ±20%. We then used the PPN to differentiate approved and unapproved plans. This was a standard classification problem, and the performance was evaluated using metrics commonly employed including accuracy, sensitivity, and specificity. We also computed the receiver operating characteristic (ROC) curve and computed the area under the curve (AUC). To further demonstrate PPNs behavior, we examined how the output of PPN depended on the input dose values. In addition, we utilized t-distributed stochastic neighbor embedding (t-SNE) to interpret PPNs classification behavior using the feature representations extracted from the second last dense layer of PPN. As a byproduct of developing PPN via the adversarial training scheme, the DPN was trained to output the physician’s desired plan dosimetric characteristics. Hence, we quantify DPNs performance by comparing its output EQD2 values with the ground truth in plans previously approved by the physician and reporting relative errors.

## 3. Results

### 3.1. Training and validation studies

**Figure 3.**
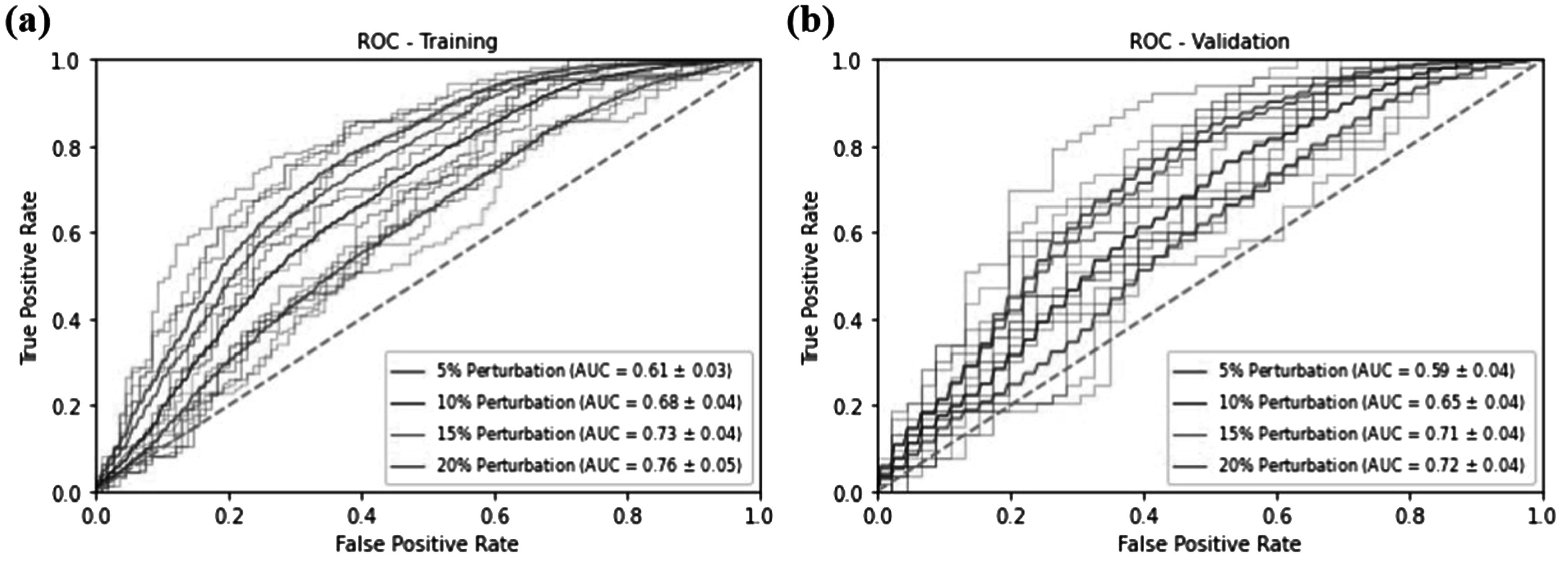
ROC curves for the (a) training and (b) validation datasets. Transparent lines represent individual ROC curves in the four-fold cross-validation study for each perturbation scaling factor. Solid lines are the mean ROC curves across four folds for each perturbation scaling factor.

Figure 3 shows the ROC curves for the training and validation data sets in terms of differentiating between approved and unapproved plans by the PPN. In both sub-figures, for each scaling factor, we first plotted four individual ROC curves for the PPN models generated via the four-fold cross-validation study. For each perturbation factor, mean ROC curves were estimated across the four folds, shown as solid lines.

Quantitative evaluation results of the four-fold cross-validation study are summarized in table 1. When differentiating approved and perturbed plans with a scaling factor of ±5%, ±10%, ±15% and ±20%, for the training data set, on average PPN achieved an AUC of 0.61 ± 0.03, 0.68 ± 0.04, 0.73 ± 0.04, and 0.76 ± 0.05. The numbers for the validation data set were approximately the same, indicating that the model was not trained to overfit data. As the perturbation scaling factor increased, it became relatively easier to differentiate plans, and hence the model performance generally increased.

**Figure 4.**
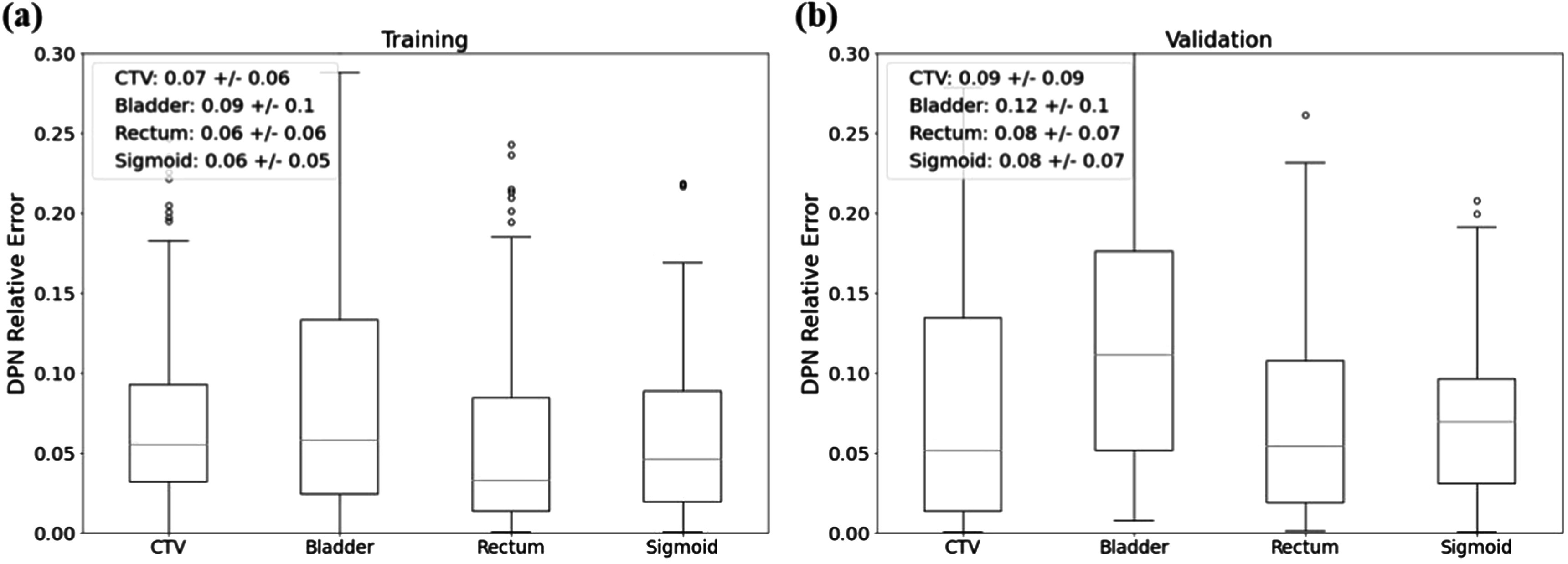
Relative errors of DPN evaluated on (a) training and (b) validation datasets. The central red mark indicates the median. The top and bottom edges of the box indicate 75th and 25th percentiles, respectively.

As for the DPN, the relative errors of the bladder, rectum, sigmoid, and CTV were 9.21% ± 9.51%, 5.86% ± 6.04%, 5.95% ± 4.18%, and 7.10% ± 5.84%, respectively, for the training data set. When evaluating the performance on the validation data set, the relative errors for the bladder, rectum, sigmoid, and CTV were 12.47% ± 10.08%, 7.55% ± 6.77%, 8.10% ± 7.27%, and 8.89% ± 8.8%, respectively. Among the four models generated, the one with the best performance was advanced to the independent testing stage. The results of the best-performed fold are shown in figure 4. It is worthwhile to remark that the dose prediction error was relatively large compared to those reported in other studies for the dose prediction task in EBRT (Chen *et al* 2019, Nguyen *et al* 2019). This can be ascribed to the fact that data quality consistency in HDRBT is lower than in EBRT, which will be investigated and discussed in the discussion section.

### 3.2. Testing studies

#### 3.2.1. PPN evaluation

**Figure 5.**
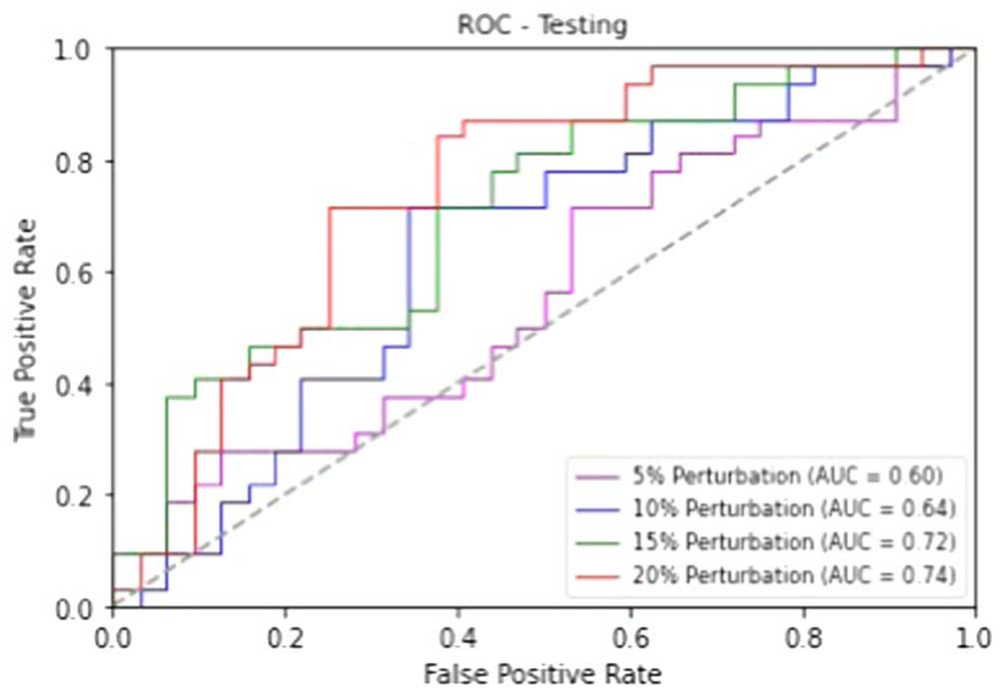
PPN analysis on the testing dataset. ROC curves for the clinical-approved plans, unapproved plans perturbed by three different levels, and the mean results.

We performed an independent test using 32 plans from 9 patients not seen by the training and validation process. Similar to the study in the training and validation data set, for each of the 32 test plans, we created extra plans considered as unapproved by randomly perturbing the ground truth EQD2 by a scaling factor. We plotted in figure 5 the ROC curve for the best-performed model in the four-fold validation study and quantitative evaluation metrics were reported in table 1. We found that the PPN was able to differentiate between approved and unapproved plans perturbed by ±5%, ±10%, ±15% and ±20% with AUC of 0.60, 0.64, 0.72, and 0.74, respectively.

**Table 1.** Quantitative evaluation, included accuracy (Acc), sensitivity (Sen), specificity (Spe), and AUC of PPN for the four-fold cross-validation study and the independent testing.

Ratio	Fold	Training				Validation				Testing			
		Acc	Sen	Spe	AUC	Acc	Sen	Spe	AUC	Acc	Sen	Spe	AUC
5%	1	0.62	0.63	0.59	0.63	0.60	0.60	0.61	0.59	0.59	0.63	0.55	0.60
	2	0.60	0.64	0.55	0.63	0.60	0.61	0.58	0.59				
	3	0.62	0.64	0.59	0.61	0.59	0.58	0.60	0.60				
	4	0.60	0.60	0.59	0.57	0.56	0.57	0.54	0.57				
	Mean	0.60	0.63	0.57	0.61	0.59	0.59	0.58	0.59				
	Std	0.03	0.03	0.02	0.03	0.03	0.03	0.03	0.04				

10%	1	0.64	0.66	0.62	0.70	0.65	0.66	0.64	0.67	0.61	0.65	0.58	0.64
	2	0.62	0.65	0.56	0.69	0.65	0.67	0.62	0.65				
	3	0.64	0.68	0.60	0.69	0.66	0.65	0.66	0.66				
	4	0.61	0.63	0.59	0.63	0.64	0.63	0.64	0.62				
	Mean	0.63	0.66	0.60	0.68	0.65	0.65	0.64	0.65				
	Std	0.03	0.02	0.03	0.04	0.02	0.04	0.03	0.04				

15%	1	0.68	0.70	0.65	0.76	0.68	0.69	0.67	0.73	0.66	0.70	0.61	0.72
	2	0.67	0.74	0.59	0.75	0.68	0.70	0.66	0.72				
	3	0.67	0.71	0.62	0.76	0.69	0.68	0.69	0.71				
	4	0.66	0.68	0.64	0.66	0.65	0.64	0.65	0.67				
	Mean	0.67	0.71	0.63	0.73	0.67	0.68	0.67	0.71				
	Std	0.03	0.03	0.02	0.04	0.03	0.04	0.03	0.04				

20%	1	0.73	0.76	0.67	0.78	0.71	0.73	0.69	0.76	0.70	0.74	0.65	0.74
	2	0.70	0.76	0.64	0.78	0.69	0.71	0.67	0.75				
	3	0.69	0.73	0.66	0.79	0.70	0.69	0.70	0.74				
	4	0.69	0.71	0.66	0.69	0.67	0.66	0.67	0.68				
	Mean	0.71	0.74	0.66	0.76	0.69	0.70	0.68	0.72				
	Std	0.03	0.03	0.02	0.05	0.04	0.03	0.02	0.04				

**Figure 6.**
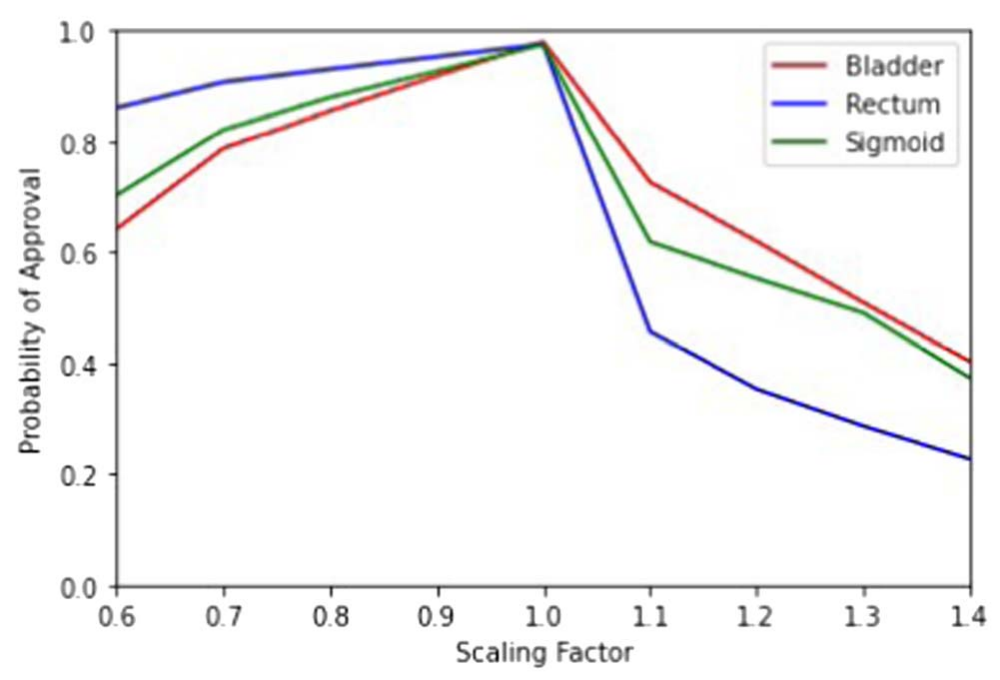
Probability of plan approval output by PPN as a function of a scaling factor applied to scale the EQD2 of the bladder (red), rectum (blue), and sigmoid (green) of the clinically approved plans.

To further examine the behavior of the PPN in detail, for each clinically approved plan, we held the *D*_90%_ of CTV unchanged and adjusted the *D*_2*cc*_ of one OAR at a time with a scaling factor ranging from 60% to 140%. We then observed the output of PPN with respect to the scaling factor, which reflects the dependence of the probability of plan approval on *D*_2*cc*_ values. The output averaged over all cases was plotted in figure 6. Specifically, the probabilities of plan approval peaked at the scaling factor of 1.0, indicating the favor of the clinically approved plans. As the scaling factor increased, the plan approval probability started to sharply reduce, as these plans became unacceptable due to overdose. In the other direction, while the scaling factor was decreased from 1.0, the probability remained relatively high within a certain range. This implied that PPN can still accept plans with dose lower than those approved clinically. In the extreme with a low scaling factor, the predicted probability started to decrease. Yet in practice, the plan with low OAR doses should be favored. The behavior of PPN can be ascribed to the following. Given that the dose is EQD2 of the entire course, scaling down the total EQD2 by, e.g. 20% while keeping the EBRT dose unchanged actually means scaling down of the dose at a fraction largely over that level. In our training data set, it is rare to see cases with such large dose reductions. As we trained the model under the assumption that all unseen plans are unapproved, the PPN tends to predict a drop of plan approval probability at this OAR dose reduction level. This issue may be addressed in future work by adding more training data including augmented approved cases with manually reduced OAR doses.

**Figure 7.**
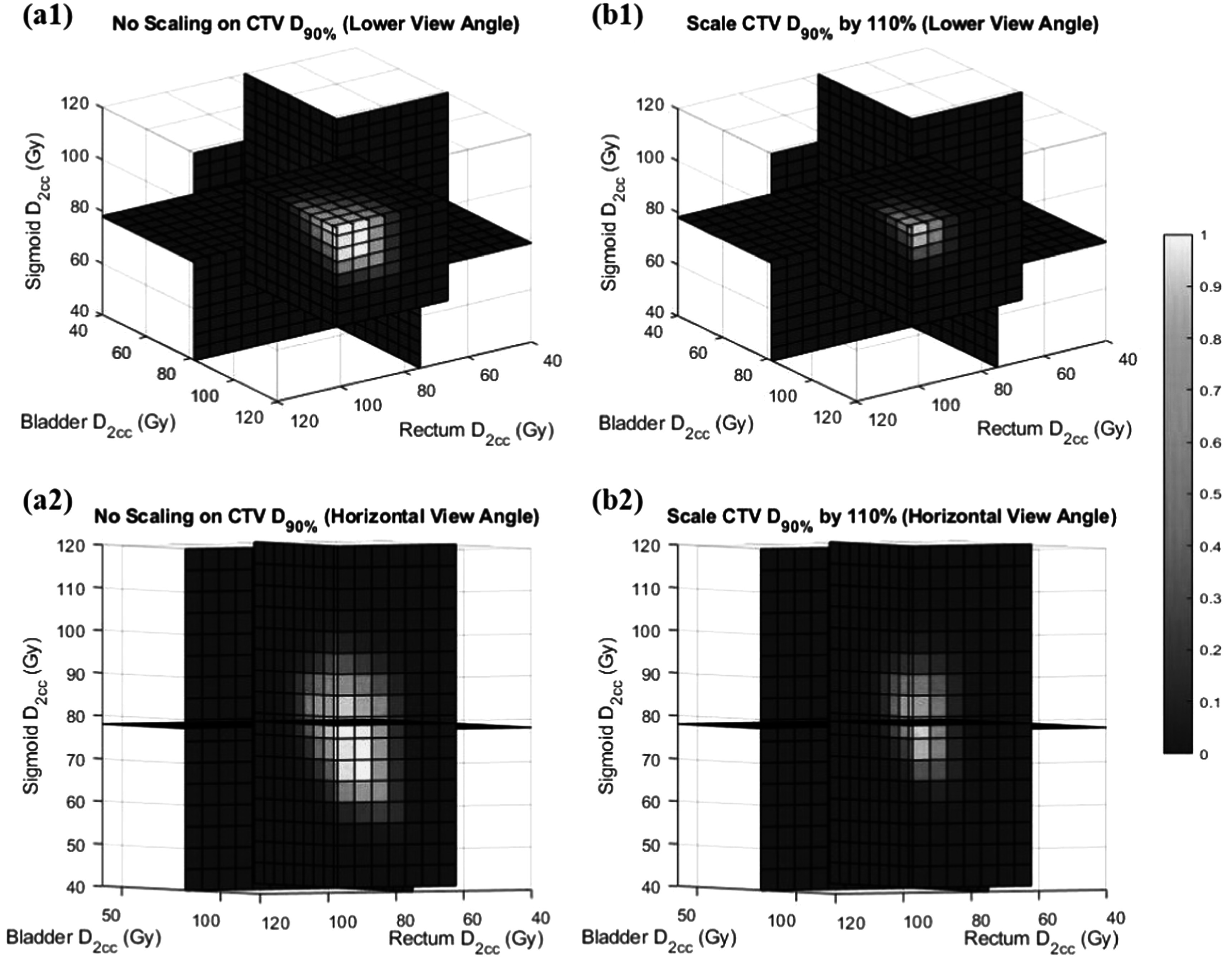
Predicted probability of plan approval for a representative case as a function of three *D*_2*cc*_ values with CTV *D*_90%_ from (a) the clinical plan and (b) scaled by 110%. Figures in the top and bottom rows are the same plots but viewed from different view angles.

Figure 7 demonstrates the behavior of PPN for a representative testing case. Specifically, in the left subfigure, keeping the CTV *D*_90%_ unchanged, we plot the probability of plan approval as a function of the three *D*_2*cc*_ values in a color scale. The intersection of the three planes corresponded to the clinically approved plan. As expected, acceptable plans with high probabilities of plan approval were clustered in the central region around the clinically approved plan. As either one of the *D*_2*cc*_ started to increase, the probability dropped sharply. In the opposite direction when the *D*_2*cc*_ decreased, the probability remained at a relatively high level but eventually dropped. We further performed the same study but scaled CTV by 110%. At a higher level of CTV *D*_90%_, with the same *D*_2*cc*_ of OAR, the probabilities of plan approval were generally decreased, which can be ascribed to over-dosed CTV.

**Figure 8.**
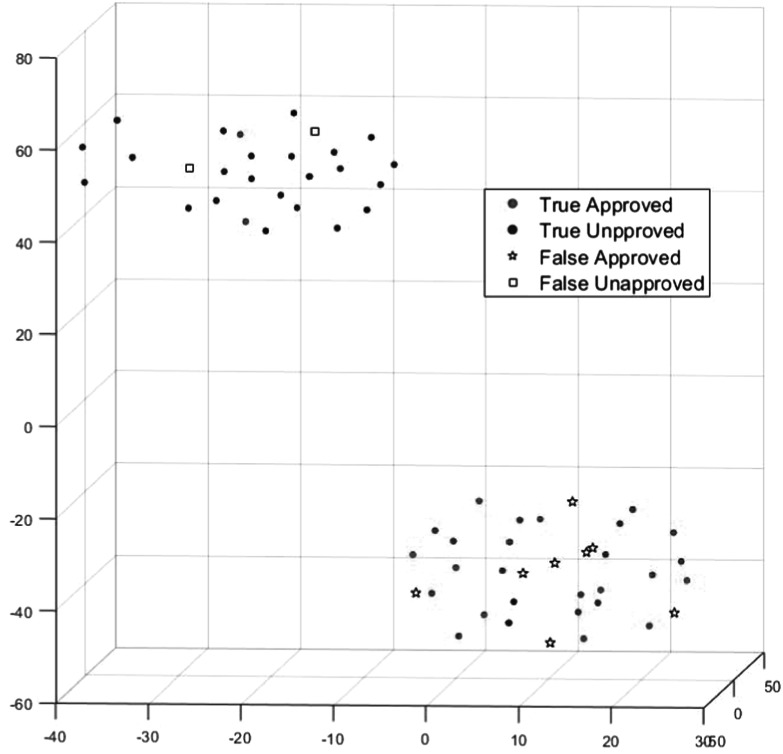
3D t-SNE plot of the feature representations extracted in the latent space learned by PPN. Red and blue dots are PPN-approved and PPN-unapproved plans that also agree with the ground truth label. Pentagram and square symbols represent plans approved and unapproved by PPN but incorrectly.

Figure 8 presents the high-dimensional feature mapping using the 3D t-SNE approach, which was able to reduce high-dimensional features extracted from PPN into three dimensions for visualization. For each approved testing case, we again created one corresponding unapproved plan by randomly perturbing the ground truth EQD2 of the bladder by 15%–25%. In the 3D space, it became clear that approved and unapproved plans considered by PPN were separated into two clusters. We noticed a few plans were predicted incorrectly by PPN and did not agree with the ground truth. This may be ascribed to the inconsistent plan quality in the collected data due to the intrinsic plan quality variations in HDRBT. Data consistency in HDRBT may be a concern that prevents the achievement of accurate model prediction.

#### 3.2.2. DPN evaluation

**Figure 9.**
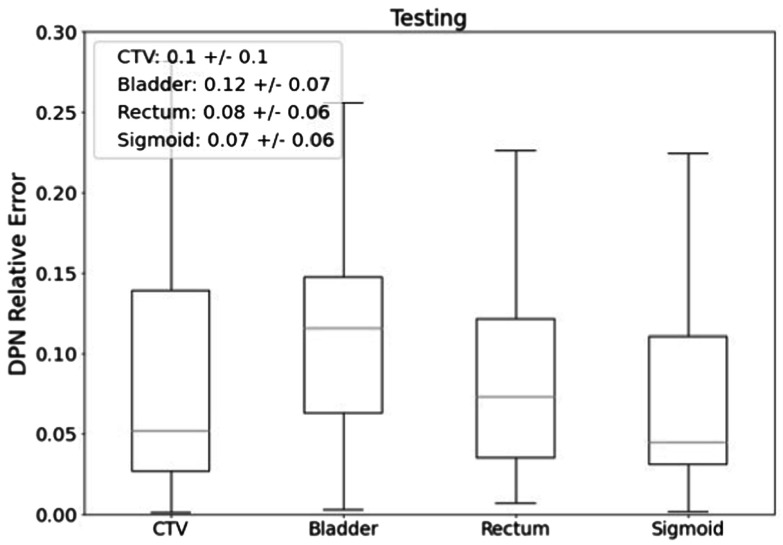
Relative errors of DPN prediction for different structures evaluated on the testing data set.

The performance of DPN was evaluated using the 32 independent testing cases. Boxplots for the predicted error of individual structures are shown in figure 9. We found that the DPN was able to achieve an average prediction error of 11.51% ± 6.92%, 8.23% ± 5.75%, 7.12% ± 6.00%, and 10.16% ± 10.42% for the bladder, rectum, sigmoid, and CTV, respectively.

## 4. Discussions

Aiming at building a metric that reflects the probability of plan approval in HDRBT and can serve as the guidance for automated treatment planning, this study constructed a PPN using the deep learning approach. In addition to this intended application, PPN may also be used to evaluate the variations of plan approval probability. It is well known that there is a subjective component when measuring plan quality and acceptability in treatment planning, e.g. driven by the physician’s training and experience. Predicting plan approval with such a component included is important to facilitate automatic treatment planning, improve standardization, and reduce quality variations.

A byproduct of constructing PPN via the adversarial learning scheme was DPN, which was established to output the physician’s desired dosimetric variables based on specific patient anatomy and prescription level. This problem has been extensively studied, although the existing studies mostly focused on EBRT and those on HDRBT are relatively rare. The DPN developed in this study may be used to provide guidance to the planner in terms of desired CTV *D*_90%_ and OAR *D*_2*cc*_ values, which potentially can facilitate the treatment planning process.

With the success of the current work, we intend to use the PPN as the guide for our DRL-based virtual treatment planner in HDRBT (Shen *et al* 2019). The DRL-based planner reported in our previous publication was trained to interact with an HDRBT optimization engine and autonomously adjust parameters in the optimization engine to maximize a reward function. The reward function was defined in the form of a weighted sum of OAR’s *D*_2*cc*_ values of different organs, which are clinically meaningful to a certain extent, but do not necessarily reflect the physicians true preference in plan approval. In particular, it only considered the OAR’s *D*_2*cc*_ values of the current fraction, whereas the total dose over the entire course was ignored. Hence, the plans generated by the virtual planner may not effectively meet the physicians requirements. We expect using the PPN as the reward function to train the virtual planner will overcome this challenge.

While our study followed a similar scheme to our previous study in prostate cancer stereotactic body radiation therapy (SBRT) (Gao *et al* 2022), aligning with our general research direction seeking quantitative measures to guide automatic treatment planning, the current study has specific contributions and novelty. First, this study focused on the unique application of HDRBT and was adapted to the decision-making setting in this context. Different from prostate SBRT plan approval based on the dose of the plan, HDRBT plan approval requires the consideration on the total dose of the entire treatment course, including that of EBRT, delivered HDRBT fractions, current fraction for approval, and expected dose for remaining fractions. We designed the model to include the required dose information and properly incorporate it into the network for decision-making. Second, the unique challenge of plan quality variation in HDRBT makes it difficult to follow the same approach in the prostate SBRT study that employed dose distribution and organ masks directly as PPN and DPN models’ input. To overcome this challenge, we employed distance histograms instead of 3D mask images to represent anatomy, and dosimetric variables rather than 3D dose distributions to represent dose, which is useful for extracting relevant information uniquely applicable to HDRBT to develop PPN and DPN.

We would like to further elaborate on the reason for using dosimetric variables rather than 3D dose distributions as input to the network models. In contrast to prostate SBRT plan evaluation, where physicians typically consider both 3D spatial information of dose distributions and dose metrics, HDRBT plan evaluation is more straightforward, mainly focusing on the four dosimetric variables, as these variables are demonstrated to be correlated with treatment outcomes (Romano *et al* 2018). Given this knowledge, it is unnecessary to input to the network the entire 3D dose distribution and rely on the network to identify features relevant to plan approval. Another reason for the choice is to directly use clinically relevant quantities for decision-making to overcome the problem caused by the large plan quality variation in HDRBT. Under the large plan quality variation, using the dose distribution as input is expected to impede the effectiveness of model training, potentially leading to overfitting.

Using DNNs in this study leverages the inherent flexibility of neural networks to approximate complex functions compared to classical machine learning methods. Particularly in the dose prediction component, there is a need for a function mapping from anatomy and prescription to dosimetric variables. While it might be feasible to employ classical machine learning tools for constructing these models, doing so would require meticulous design of model functions. Therefore, we opted for DNNs to ease the model construction part. With this consideration, as well as the consideration of the relatively low data quality in HDRBT, we attempted to maintain a relatively small scale for the networks to avoid model overfitting, as opposed to using large-scale DNN models in our previous study on prostate cancer SBRT. Specifically, we conducted experiments involving various widths and depths of the PPN and DPN to find a balance between model performance and complexity, ensuring optimal performance of our models while mitigating the risk of overfitting.

**Figure 10.**
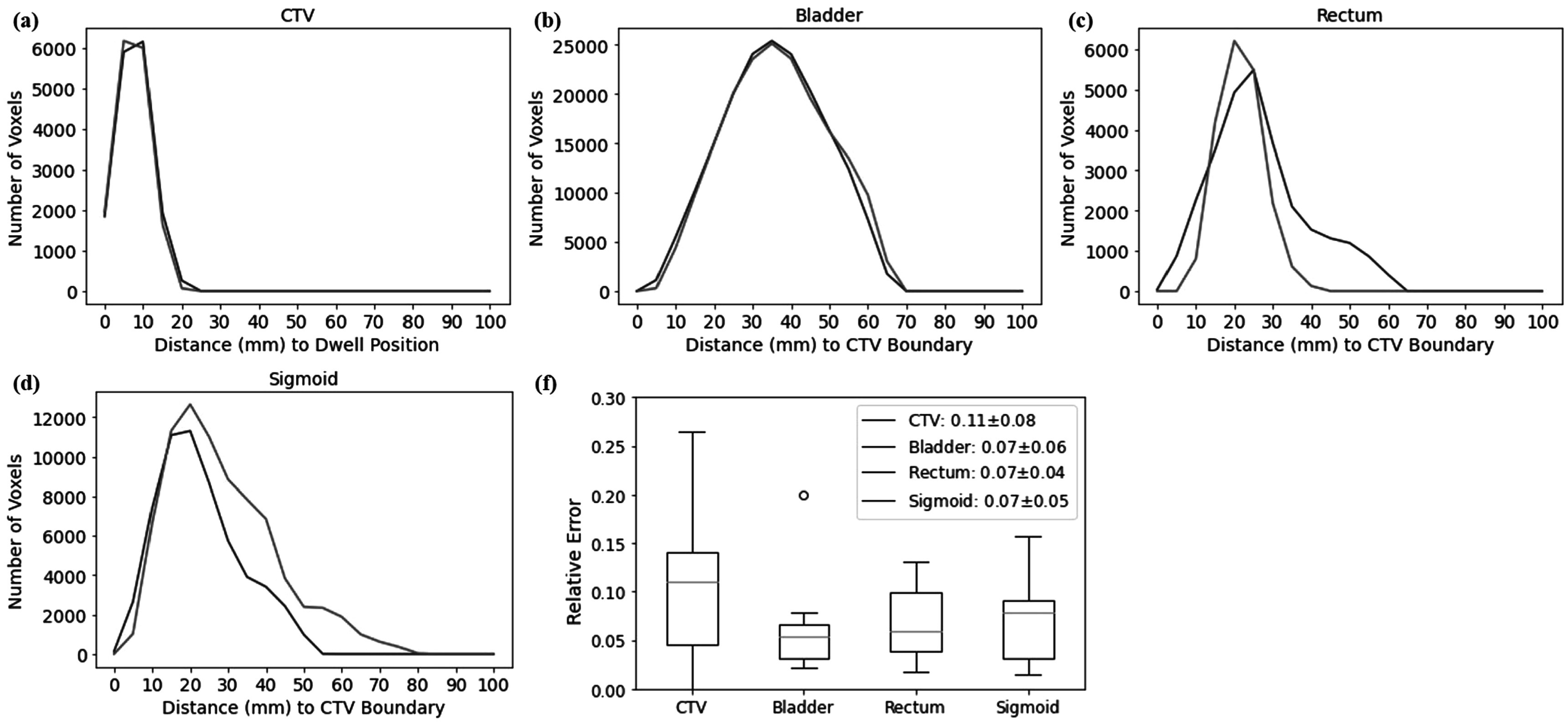
(a)–(d) Comparison of the distance histograms of CTV, bladder, rectum, and sigmoid for two cases considered similar. (b) Dosimetric variations among all cases with similar distance histograms.

The current study has the following limitations. First, being a data-driven study, the results are always limited by issues associated with data, such as data size and quality. One particular limitation is data quality consistency. Specifically, since the goal of PPN is to predict the probability of plan approval, consistent plan quality in the data used to train models is essential. To investigate this issue, we compared every pair of cases in our data and measured differences of distance histograms *d*_*i*,*j*_ = ∣*A*_*i*_ − *A*_*j*_∣, where *i* and *j* are case indices and ∣.∣ is matrix Frobenius norm. We identified pairs that are considered similar for *d*_*i*,*j*_/(∣*A*_*i*_∣ + ∣*A*_*j*_∣) < 10% and case *i* and *j* have the same prescription. The threshold of 10% was chosen empirically for this analysis. Figures 10(a)–(d) illustrate two distance histograms that were considered similar. As an example, we identified 6662 pairs of similar cases, all with prescription dose of 550 cGy per fraction. We computed relative differences in CTV *D*_90%_ and OAR *D*_2*cc*_ of each pair. Figure 10(e) presents the results in a box plot. It was found that dosimetric variations for the bladder, rectum, sigmoid, and CTV were ∼10%. Note that all the cases here were clinically approved plans. This level of variations of dosimetric measures among similar cases indeed poses a limitation in terms of accuracy that a data-driven model can achieve. The dose prediction accuracy of DPN (e.g. figure 9) was of the same level, indicating that our model has been trained to reach this limit. Since DPN and PPN are trained together, the limitation in DPN accuracy would inevitably be transferred in PPN accuracy. The reason for this variation in data may be multifolds, for instance, physicians’ experience, time available for decision-making in treatment planning, etc. While this fact poses a challenge to build a model to predict the chances of plan approval, it also implies the importance of this study, as having a plan approval prediction model and implementing it in treatment planning is expected to reduce the variation.

Second, in fact, it is difficult, if not impossible, to find the ground truth label for a plan in terms of being approved or unapproved. For the real clinical data, even a clinically approved plan may not be truly approved, in terms of achieving the best possible quality. It could be possible that the plan was approved under the time pressure of the busy clinic, although there is room to further improve its quality. Meanwhile, the unapproved plan may not be truly unapproved either. The data available for our model development are all collected from routine clinical practice, which by default are all approved plans. Those unseen plans were simply considered unapproved in this study. Yet it could be possible that the busy clinic did not allow careful plan tuning to generate a high-quality plan, and the unseen, potentially high-quality plan, should be approved. It is worth mentioning that this situation is much worse in HDRBT than EBRT due to busy clinical operations. With these considered, it is difficult to delineate clear boundaries between approved and unapproved plans, posing challenges both for model development and evaluation. Without ground truth labels, it is not possible to perform a rigorous test of the PPN model. Nonetheless, we expect that the approval probability remains high within a certain range around the clinically approved plan but decreases as we move away from this. Therefore, we demonstrated this aspect to show the reasonable performance of the PPN model, rather than for a rigorous test of its performance. The 5%–20% perturbation levels were empirically chosen for this purpose. As expected, the smaller the perturbation was, the more blurred between approved and unapproved plans, making the classification performance of the trained model worse. The same behavior can also be verified in figure 7.

## 5. Conclusion

In this paper, we developed a deep-learning method that can predict a probability to approve HDRBT plans of cervical cancer. We achieved this by jointly training a DPN to predict patient-specific dose and a PPN that predicts the approval probability of a plan. Quantitatively, DPN predicted EQD2 with a relative error of 11.51% ± 6.92%, 8.23% ± 5.75%, 7.12% ± 6.00%, and 10.16% ± 10.42% for bladder, rectum, sigmoid, and CTV, respectively. In a task to differentiate clinically approved plans and disapproved plans generated by perturbing doses in ground truth approved plans by 20%, PPN achieved accuracy, sensitivity, specificity, and area under the curve 0.70, 0.74, 0.65, and 0.74. To our knowledge, this is the first time a method has been developed to predict a plan approval probability in HDRBT treatment planning. The developed PPN is expected to be a useful tool in guiding the automated treatment planning process to generate plans favored by physicians.

## Data Availability

The data cannot be made publicly available upon publication due to legal restrictions preventing unrestricted public distribution. The data that support the findings of this study are available upon reasonable request from the authors.
